# Factors Influencing the Load-Bearing Capacity of Rock as Base Material for Post-Installed Anchors

**DOI:** 10.3390/ma14185130

**Published:** 2021-09-07

**Authors:** Stefan Lamplmair, Oliver Zeman, Klaus Voit

**Affiliations:** 1 Institute of Applied Geology, Department of Civil Engineering and Natural Hazards, University of Natural Resources and Life Science (BOKU), 1190 Vienna, Austria; Stefan.Lamplmair@gmx.at (S.L.); klaus.voit@boku.ac.at (K.V.); 2 Institute of Structural Engineering, Department of Civil Engineering and Natural Hazards, University of Natural Resources and Life Science (BOKU), 1190 Vienna, Austria

**Keywords:** base material, rock, post-installed anchors, adhesive anchor, mechanical anchor, load-bearing capacity, GSI, RMR, rebound value, rebound hammer

## Abstract

In the case of fastenings on rock, as a result of the variability, it is quite difficult to make a preliminary assessment of the load-bearing capacity of rock as a base material. This paper therefore investigates which rock parameters next to an anchor position have an influence on the load-bearing capacity. For this purpose, tests are carried out on post-installed anchors in different lithologies in eastern Austria. It can be shown that the joint weathering has an influence on the load-bearing capacity of post-installed anchors and conclusions can be made about joint weathering by means of rebound hammer. Rebound values can therefore also be used to draw conclusions about the rock quality as a base material for post-installed anchors. Nevertheless, a combined optical assessment of the base material is recommended as an adequate method.

## 1. Introduction

Concrete and masonry as base materials for post installed anchors are well considered by different regulations [1,2]. For concrete, in [3], the load-bearing behaviour was investigated and transferred into a design concept. The load-bearing behaviour and the design of bonded anchors were investigated by means of numerical investigations in [4]. More detailed numerical investigations of bonded anchors were carried out in [5]. Therefore, for engineers drawing conclusions about the load-bearing behaviour and capacity in advance, the base material is predefined and well known. In contrary, intact rock (e.g., rock slopes or walls) as a base material is not considered by regulations, not least because of the numerous possible variations of the rock properties. A large number of different installation parameters, such as existing joints, joint condition, uniaxial compressive strength, or weathering degree, induce uncertainty regarding the rock quality. In other words, due to the inhomogeneity of rock, these varying factors lead to insufficient information about the behaviour of post-installed anchors. Therefore, a number of experimental investigations concerning the load-bearing behaviour and capacity of post-installed anchors in rock have been performed in [6,7,8]. Contrafatto and Cosenza [6,7] were able to investigate the behaviour of chemical anchors in different rock types in the laboratory [6] and to test the applicability of concrete prediction models for natural stone [7]. For this purpose, anchors were installed in blocks of different rock types in the laboratory and tested until failure. Three embedment depths were selected depending on the anchor diameter. The aim was to define the minimum embedment depth at which steel failure occurs, since this can be determined by calculation. Subsequently, theoretical models for determining the load-bearing capacity in concrete were checked for their applicability to natural stone. According to Contrafatto and Cosenza [6,7], the applicability is only given if the mechanical rock characteristics are similar to those of concrete.

Tóth et al. [8] were able to identify the compressive strength, flexural strength and porosity as the main influencing factors, although the rocks investigated in [8] appeared to be relatively homogeneous rock types. It should be noted that the investigations in [6,7,8] were carried out in a laboratory environment using prepared blocks containing few joints. Hence, it must be assumed that the rock properties are more homogeneous than in a non-laboratory environment. Therefore, the investigations in [6,7,8] are considered to be relevant for natural stone masonry. To further consider inhomogeneous characteristics in intact rock, in [9], the authors examined different geologies in Eastern Austria in a non-laboratory environment to gain experience when considering the load-bearing capacity of anchors. From this contribution [9], the following main conclusions can be drawn:

- the suitability of rock as base material for post-installed anchors can be assessed by rock classification systems;
- for assessing the load-bearing capacity, a small-scale investigation in the area of the intended fastening area is necessary;
- the presence of joints as well as their condition, such as weathering, influences the load-bearing capacity;
- in the case of poor rock quality, merely base material failure could be observed rather than failure of the anchor;
- in terms of good rock properties, which means fewer inhomogeneities, a variety of failure mechanisms have been found;
- it can also be derived that the load-bearing behavior is influenced mainly by rock properties, rather than the anchoring system;
- disturbed fastening areas (influenced by joints) show analogies to cracked concrete, while undisturbed ones (no influence by joints) behave comparable to non-cracked concrete;
- similarities between the failure mechanisms of rock and concrete were observed.

The conclusions from [9] did not consider further investigations of rock properties in the immediate vicinity of the anchor position and this remains as an open question. Also rock compressive strength and joint condition are expected to have an influence on the load-bearing capacity.

Therefore, this article examines the following research questions for intact rock including inhomogeneities: (i) Which small scale influencing parameters (e.g., rock compressive strength, joint quantity, joint weathering) have an impact on the load-bearing capacity of post installed anchors? (ii) Is it possible to find a method to determine areas with good rock quality for post installed anchors in advance? For this purpose, experiments on post-installed anchors in rock for small embedment depths were performed. In parallel, the mechanical rock properties were investigated in detail to determine the correlation between the load-bearing capacity and the mechanical rock properties. These experiments were performed in different geologies in Eastern Austria, focusing on small-scale rock parameters, like point load index, rebound values, compressive strength, and joint properties.

## 2. Materials and Methods

### 2.1. Test Program

**Figure 1:**
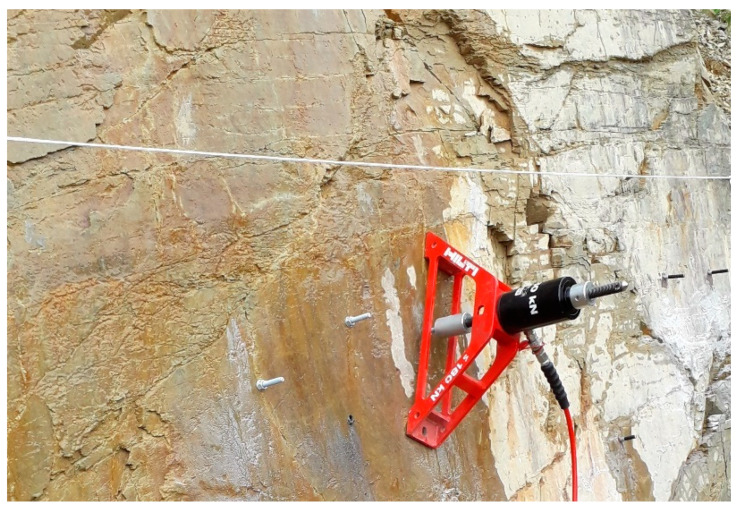
Test setup with hydraulic handpump, wide load bridge and scanline.

The conducted test campaign is listed in Table 1 and considers different testing parameters. The test program has already been used to investigate how post-installed anchors with low embedment depths behave in rock and which rock parameters have a significant influence on the load-bearing behaviour and capacity [9]. In addition to the previous evaluations in [9], this paper shows how small-scale investigations can be used for an a priori estimation of the anchor behaviour. As Table 1 indicates, the test program is divided in geological investigations for rock classification of the different geologies and small-scale investigations of the rock in the immediate vicinity of the anchor position. In total, 100 pull-out tests were performed in different geologies in Eastern Austria. Of these, 55 tests were conducted in disturbed (influence by joint given) and 45 were conducted in undisturbed (no influence by joints expected) base material. Injectable adhesive anchors as well as mechanical bolt anchors with an effective embedment depth of 70 mm and a rod diameter M12 were used. Small-scale investigations carried out next to the anchor position are listed in the last row of Table 1. Pull-out tests were carried out using a hydraulic handpump without displacement measuring, as shown in Figure 1. In order to include the load bearing behavior of the rock, a wide support bridge was used.

### 2.2. Examined Geology

Geological characterization was performed on the geologies described in Table 1 and is described in detail in Section 2.2.1.

#### 2.2.1. Engineering Assessment of Examined Geology

In order to understand the load-bearing behavior and capacity of post-installed anchors in rock the experimental campaign as listed in Table 1 was performed. For rock mass classification (rock mass quality) scanlines were carried out (see upper part of Figure 1). Scanlines are performed by determining joint location, quantity, condition and orientation whereby the rock structure is recorded in detail. These data are subsequently used as input parameters for the rock mass rating (RMR) according to [17] and the geological strength index (GSI) according to [18]. The RMR represents a rock mass classification system, in which rock strength, joint distances and conditions, in addition to water influence are included as parameters. The GSI serves as a rock classification system based on a visual survey, in which rock structure and surface conditions are assessed. Considering this, deformability and rock strength can be estimated. Scanlines and the resulting rock mass classifications RMR and GSI allow a comparison between different rock types. Scanlines were performed also to record the joint structure (joint frequency, roughness, etc.). The uniaxial compressive strength (UCS) was estimated indirectly by a rebound hammer “Schmidt hammer” (Type N) according to [19] and the point load index according to [20]. Furthermore, the uniaxial cylindrical compressive strength was also determined on diamond drilled cores from the specific study area. In Table 2, the results from the above described testing program are listed. These are reflected in the rock mass quality, which represents the potential of the rock being used for fastenings [9]. In other words, it represents the best-case load-bearing capacity of the base material. Taking into account the findings from [6], it remains uncertain which small scale rock parameters are influencing the load-bearing capacity of post installed anchors. In order to improve the understanding of these parameters, small-scale investigations of rock parameters next to the anchor position were also performed.

In a first step, the previous the pull-out test rebound values (R) around the anchor position were measured, concluding the influence of rock strength. As stated by [21], rebound values indirectly describe the rock compressive strength. According to [22], 1.5 × h_ef_ is considered to be decisive for determining the tensile load-bearing capacity of anchors in concrete. Hence, rebound values were taken at a distance of 1.5 times the effective embedment depth (1.5 × h_ef_). In the following, “R” is used for the measured rebound values. As mentioned above also joint weathering and quantity are considered to have an influence on the load-bearing capacity [9]. Therefore, after performing pull-out tests, joint weathering and the quantity of critical joints were determined.

#### 2.2.2. Selection of Anchor Positions

**Figure 2:**
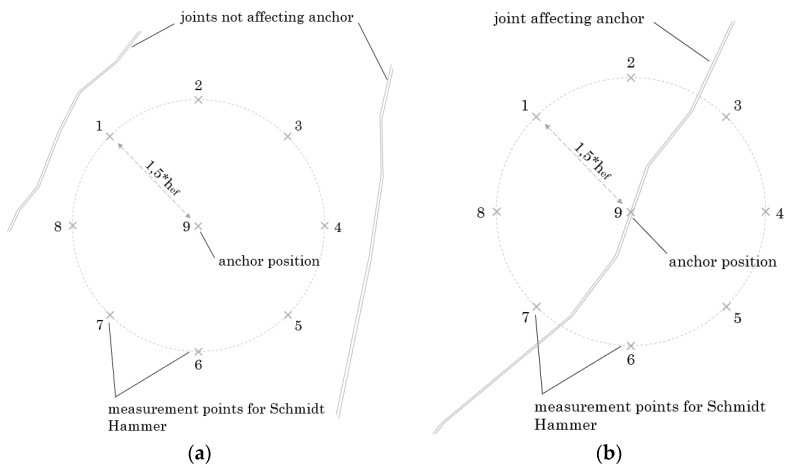
Anchor positions one to nine in (**a**) undisturbed area (not affected by visual detectable joints) and (**b**) disturbed area (affected by joints).

It is assumed that for each rock type in the investigated section there are areas which are visually on the first visual inspection better and others which are worse suited as base material (Figure 2). Well suited or undisturbed areas were assumed to be (i) not disturbed in the close-up range (1.5 × h_ef_) by visually recognizable joints (see Figure 2a). In contrast, worse suited or disturbed areas were defined as (ii) affected by at least one optically detectable joint. (Figure 2b). In order to consider the worst case in terms of fastening, anchors were directly positioned in a visually recognizable joint (Figure 2). Test quantities per rock type and fastening area (disturbed/undisturbed) are plotted in Table 1. This procedure is used for a visual assessment on site and is intended to represent two possible base material extremes and, derived from this, the fastening quality.

## 3. Results

### 3.1. Influence of the Fastening Area (Disturbed/Undisturbed)

Table 3 shows that existing joints in the fastening area next to the anchor strongly influence the load-bearing capacity. For example, granulite and granite show a deviation—undisturbed to disturbed—of approximately 60% and dolomitic limestone shows approximately 50% (see Table 3). Undisturbed areas do not show any rock disturbances due to inhomogeneities and existing joints and are therefore more suitable ex ante as a base material for fastenings.

### 3.2. Influence of Joints on the Load-Bearing Capacity

#### 3.2.1. Joint Quantity

**Figure 3:**
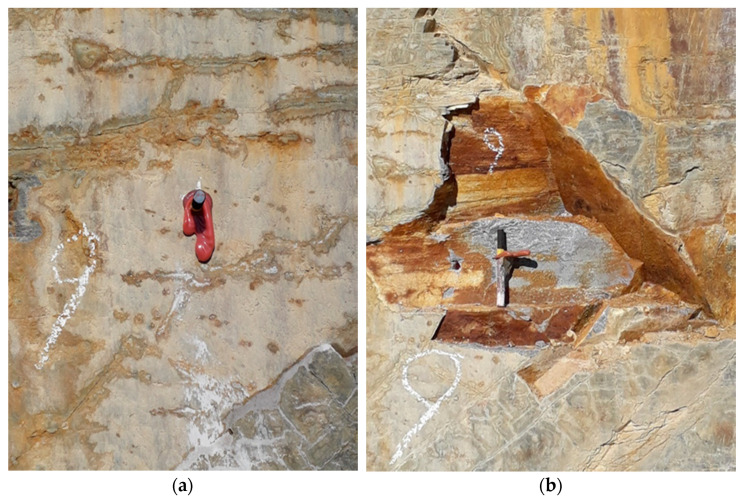
Joint quantity next to the anchor (**a**) before pull-out test in undisturbed area–no critical joints visible and (**b**) after pull-out test–critical joints visible.

As explained in Table 1, the number of critical joints was determined. The assessment was done visually after failure of the anchor occurred. It was assumed that high joint quantity results in low load-bearing capacities of the anchorages. For undisturbed areas generally a critical joint quantity of zero and high load-bearing capacities have been found. For disturbed areas, a joint quantity of at least one and low load-bearing capacities were recorded. In [9], more information about disturbed and undisturbed areas and also a comparison to classification models can be found. Thus, an influence of joint quantity on load-bearing capacity could be stated. However, further investigations of this relationship were not carried out, because critical joints can only be determined after failure occurs. Test No. 9 in granulite (epoxy resin mortar) in undisturbed base material serves as an example (Figure 3). While an optical assessment indicated an undisturbed area and no critical joints, retrospectively, the results indicated that many joints were found to be critical for failure.

#### 3.2.2. Joint Weathering

**Figure 4:**
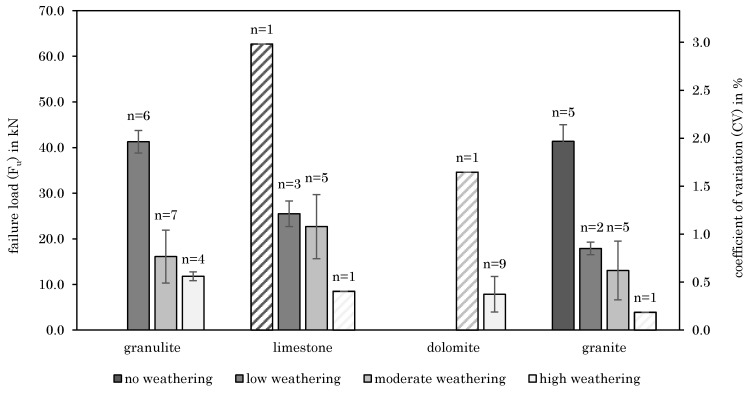
Failure load and CV as function of weathering class and rock type–weathering class determined after failure occurred (if *n* = 1 no CV is given and the beams are hatched as the data can contain an outlier).

The influence of joint weathering was considered in accordance to [23] and is mostly presented as discoloration of the surface (e.g., rust-brown coloration due to oxidation for granulite). Figure 4 demonstrates the failure load (F_u_) unrelated to the anchor type per defined weathering class and rock type. According to Figure 4, the failure load and degree of weathering are strongly related. High weathering of joints leads to lower load-bearing capacities. The secondary y axis of Figure 4 shows the coefficients of variation (CV).

**Figure 5:**
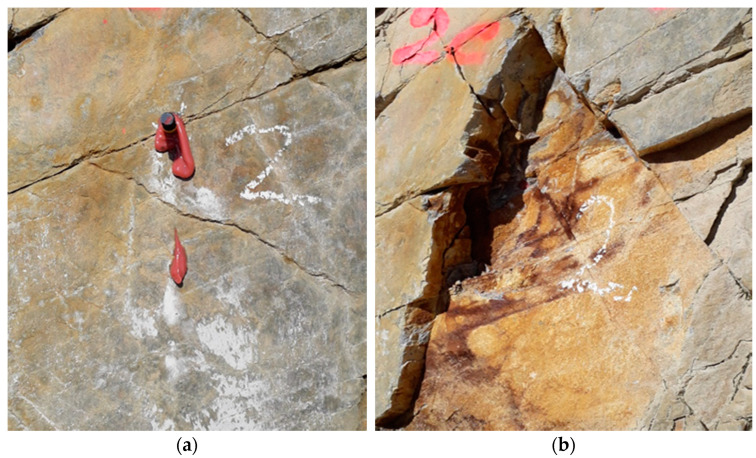
Joint weathering for (**a**) an installed anchor–weathering of joint unclear and (**b**) an anchor after failure–weathering of joint visible after failure occurred.

Comparing the coefficients of variation, it was found out that with low degree of weathering failure loads are not only higher but are also less scattering. Like joint quantity, joint weathering was determined after failure occurred, too. Figure 5 indicates the reason for that, showing an installed chemical anchor where the degree of weathering of the joint is unclear before the pull-out test is carried out (Figure 5a), although high weathering of the joints could be observed after failure occurred (Figure 5b).

**Figure 6:**
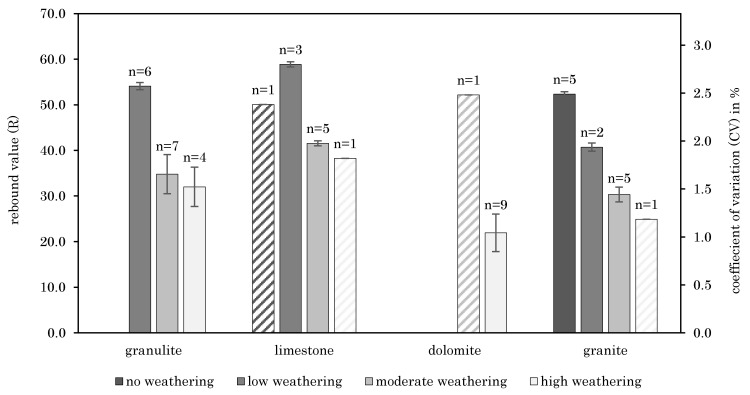
Relation between joint weathering and R including CV (if *n* = 1 no CV is given and the beams are hatched as the data can contain an outlier).

Figure 6 shows the relationship between the determined rebound value and joint weathering based on the different geologies. For high joint weathering, small rebound values were found. For low weathering, high rebound values could be determined. For different rock types, the above-mentioned correlation is varying. It should be mentioned the correlation can be determined only qualitatively, as classification of the weathering degree was also performed qualitatively. The secondary y axis of Figure 6 shows the coefficients of variation (CV). Table 4 presents the standard deviations for Figure 4 and Figure 6.

### 3.3. Base Material Assessment by Rebound Hammer

**Figure 7:**
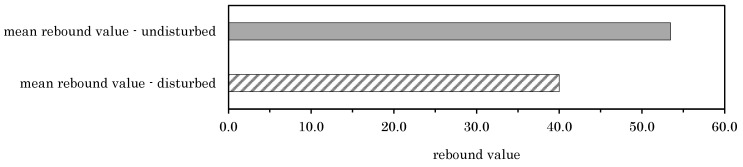
Mean rebound values for disturbed and undisturbed areas for all rock types.

As explained in Section 2.2.2, the anchor positions in the experimental campaign were first visually divided into disturbed and undisturbed areas. After this visual assessment, eight rebound values were determined circularly around the anchor position at a distance of 1.5 × h_ef_ as shown in Figure 2. Initially, rebound values were taken to provide an indication of the rock strength. Secondly, rebound values also are used to validate the classification into disturbed and undisturbed areas. As shown in Figure 7, disturbed areas lead to smaller rebound values with visible joints in the investigated area, whereas undisturbed areas show higher rebound values with no visible joints.

## 4. Discussion

### 4.1. General

The aspects discussed in the following are based on the experimental investigations described above, whereby no numerical insights are presented.

**Figure 8:**
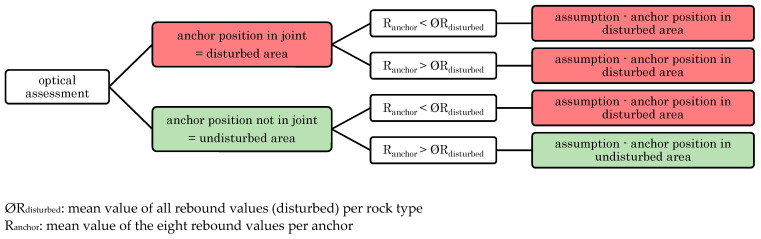
Flow chart for base material assessment by rebound hammer.

The given example in Section 3.2.1 (see Figure 3) demonstrates the difficulty of existing critical joints, which can be recognized only after failure has occurred. The fact that in some cases critical joints are not visible in advance implies that other methods in addition to a visual assessment should be carried out. A suggestion on a method how to verify the visual assessment can be found in Figure 8.

As for the joint quantity (see Section 3.2.1) the same is true for joint weathering (see Section 3.2.2). While the degree of weathering is unclear before the pull-out test is carried out, it can be easily determined retrospectively after the conduction of the test. From Section 3.2.2 it can be concluded that a low degree of weathering is well suited as an indicator for assessing the base material quality. Although a strong correlation was observed, an exact preliminary assessment of joint weathering is not possible. Hence, also the relation between the rebound value and joint weathering was investigated in Figure 6. Figure 6 indicates that joint weathering also correlates with rebound values taken around the anchor position (see Figure 2 and Section 3.2). In other words, rebound values determined next to the anchor position indicate joint weathering and thus also the base material quality. This causes a significant advantage, as rebound values can be determined easily using the base material surface, prior to failure and even before the installation of the anchor. Therefore, the relationship between rebound values and load-bearing capacities was investigated.

In order to compare rebound values with failure loads, a validation of the optical assessment was performed according to Figure 8. Firstly, the mean value of all rebound values of disturbed areas was calculated (ØR_disturbed_). ØR_disturbed_ was then compared to R_anchor_, which is the mean value of rebound values per anchor. This procedure, according to Figure 8, is used to validate the visual assessment. To be able to assume an undisturbed area, both the optical assessment and the comparison of R_anchor_ with ØR_disturbed_ must provide the result “undisturbed”.

### 4.2. Relation between Rebound Value and Load-Bearing Capacity

**Figure 9:**
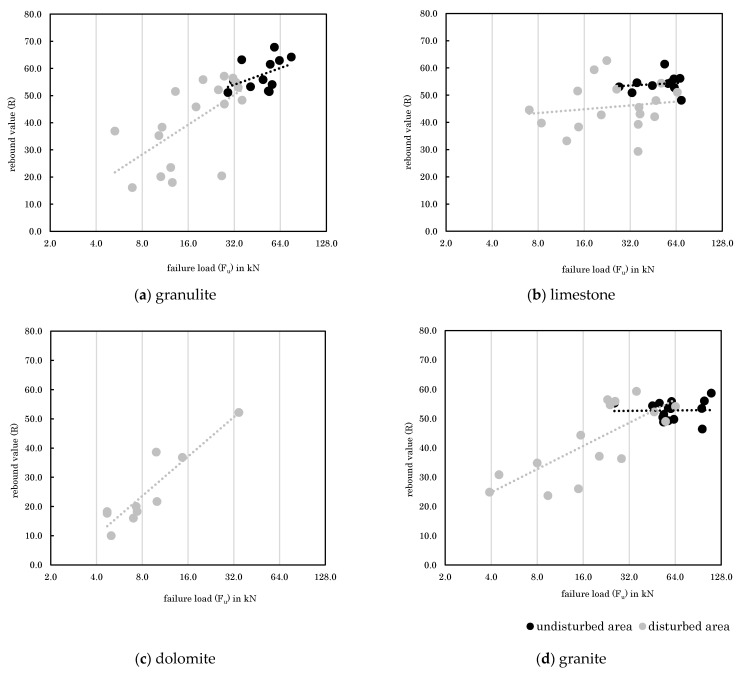
Relationships between rebound values and failure loads for various rock types.

As concluded in Section 3.1 it seems that rebound values next to the anchor position can be used as an indicator for rock quality as base material. Therefore, Figure 9 plots the relationship between failure load and rebound value (as the mean value of 8 values circularly around the anchor position) per rock type and fastening area (disturbed/undisturbed). According to Figure 9, the disturbed areas (grey color in Figure 9) are characterized by lower rebound values and failure loads, while the values are widely distributed around the line of closest fit. In contrast, undisturbed areas (black color in Figure 9) show high rebound values and high failure loads for all rock types. It can also be seen that disturbed and undisturbed areas cannot be clearly separated, but on the contrary are overlapping to some extent. Considering this in addition to the existing relationship between rebound values and failure loads, it can be concluded that the estimation of base material quality by determining rebound values is possible. However, it should be noted that a high correlation is evident for disturbed and not for undisturbed areas. For the undisturbed areas, the rebound values are capped at about 70 while the failure loads are not. The lack of correlation demonstrates that the rebound hammer is not indicative for the failure load above a certain rock strength but ensures a minimum value of load bearing capacity. The exponential correlation curve between rebound values and uniaxial compressive strength [19] is considered reasonably low for the undisturbed areas. In addition, it is possible that microcracks in the undisturbed rock cannot be detected by means of rebound hammers, although they have a significant influence on the loading of the anchor pull-out, since here the rock experiences tensile stress. For dolomite, no undisturbed fastening areas could be observed, and therefore only one data set for disturbed areas is shown. Accordingly, estimating base material quality by rebound values varies in its suitability for different rock types.

**Figure 10:**
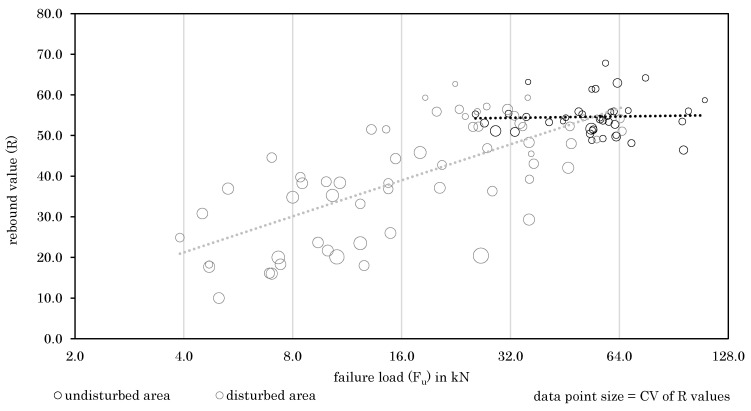
Relationship between rebound values and failure loads for all rock types.

In Figure 10, all rock types are examined together. The rebound values (R) illustrated are equivalent to the mean rebound values from the anchor position. These are calculated out of eight values taken next to the anchor position (compare to Figure 2). Therefore, it is possible to indicate not only the mean rebound values, but also to plot the scattering per value (described by the coefficient of variation, CV). This form of presentation was chosen in Figure 10, where large data points indicate a high coefficient of variation. According to Figure 10, disturbed areas result in more scattering of rebound values than undisturbed areas. Inhomogeneities around the anchor position in disturbed areas thus lead to the varying rebound values. In undisturbed areas, intact rocks with more homogeneous properties lead to a more uniform distribution of rebound values, whereas low CVs should therefore indicate good rock properties.

**Figure 11:**
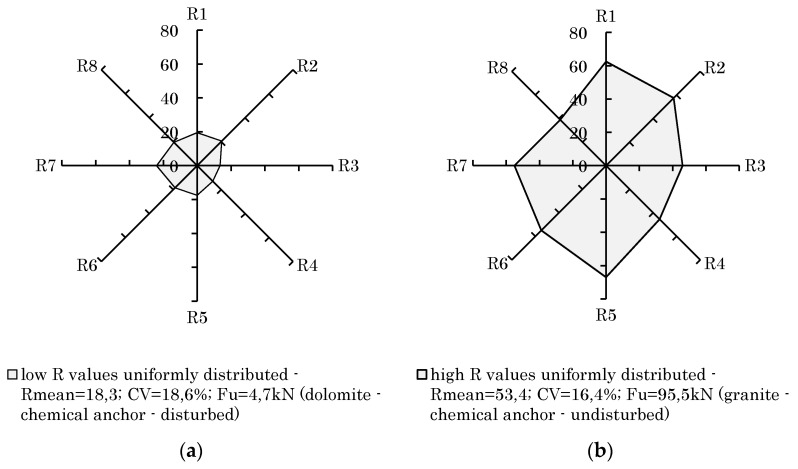
Uniformly distributed rebound values R (CV, Rmean, F_u_). (**a**) uniformly distributed with low R values; (**b**) uniformly distributed with high R values.

On the contrary, poor uniformly distributed rebound values can lead to a small scattering too. Small CVs therefore do not automatically indicate good fastening properties. Instead of that, they can originate from good or poor a priori rock properties. In Figure 11, the measured rebound values taken next to the anchor are shown using a radar chart. For both examples, the rebound values are evenly distributed. Although this results in similar CVs, the failure loads differ significantly. Therefore, using CVs to identify good rock properties is not sufficient. In order to prevent wrong conclusions being drawn from a low CV in regard to good rock properties, it is necessary to have a combined consideration of CV and mean R value.

**Figure 12:**
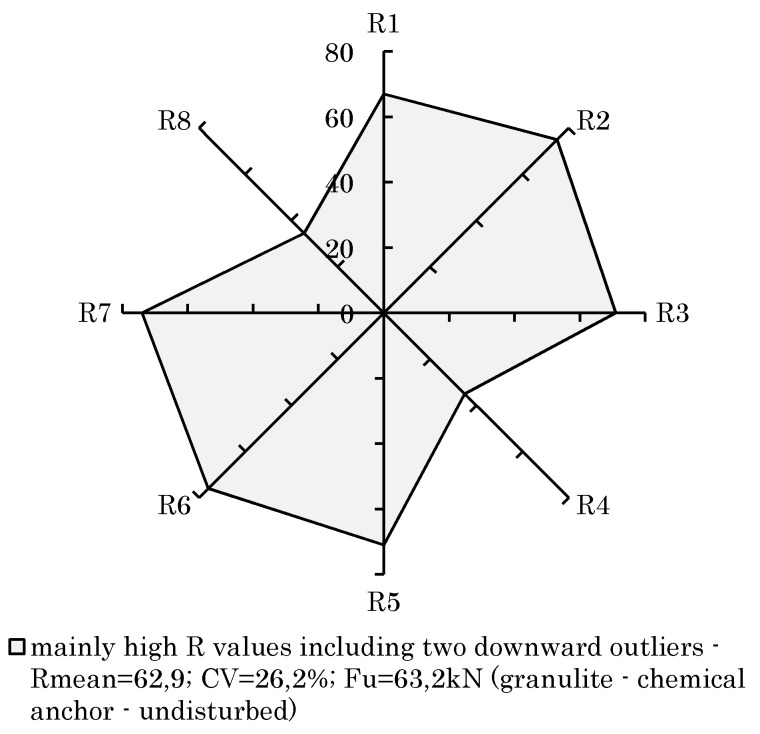
High rebound values including two downward outliers (CV, Rmean, F_u_).

Thinking further, it is also possible to neglect the CV altogether. In fact, the rebound value scattering is not decisive if the mean rebound value succeeds a certain level. Figure 12 demonstrates an example for this conclusion. Although a high CV can be observed due to downward statistical outliers, it was still possible to achieve a good load-bearing capacity. Hence, in order to identify good rock properties in advance, it should be sufficient if the tested rebound values per anchor are not falling below a certain level. A further consideration of the coefficient of variation was therefore not carried out.

**Figure 13:**
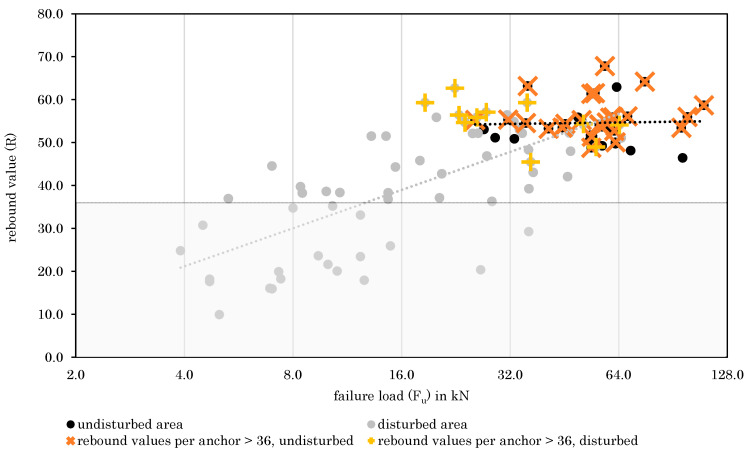
Relationship between rebound values and failure loads–anchors where R values do not fall below threshold are highlighted.

In Figure 13, a lower threshold value for the rebound values was defined. In order to determine the threshold value, a log normal distribution was assumed for all undisturbed rebound values. Calculating the 5% fractile from this distribution, a mean rebound value of 53.2, a standard deviation of 1.3, and a k-value of 1.645 for an infinite sample size were used. Conclusively, a threshold of 36 resulted. When highlighting all anchors in undisturbed areas for which none of the eight rebound values is below this threshold, good rebound values and failure loads can be obtained.

Some data points for disturbed areas also show comparable failure loads and rebound values. In principle, the same procedure can be used for anchor positions in disturbed areas, if compared to the threshold of undisturbed rebound values. However, resulting failure loads are lower and show a larger scattering, which can be explained by the existing inhomogeneities in the base material. The rock quality for post-installed anchors for these areas could be described as average.

The approach should therefore always be used in combination with a visual assessment. Nevertheless, Figure 13 indicates that using a lower threshold allows to identify good rock properties when combined with a visual assessment. A classification of base material properties by means of a combination of visual assessment and the determined threshold can be obtained from Table 5.

As described above, good base material quality can be assumed when the threshold is met in undisturbed areas. Average base material can be expected if (1) the rebound values are above the threshold in disturbed areas or (2) the threshold is not fulfilled in undisturbed areas. Ultimately, poor base material should be considered when rebound values are below the limit value in disturbed areas.

## 5. Conclusions

Small scale investigations on rock were performed in order to identify influencing parameters on the load-bearing capacity of post-installed anchors. It was shown that, for disturbed areas, a high number of joints are usually critical for failure and lead to low failure loads. For undisturbed areas, higher load capacities could be found with fewer joints, whereby the joint quantity was determined retrospectively. Nevertheless, it was shown that it is not possible to determine critical joints before failure occurs.

Weathering degree also shows a correlation with failure loads. High degrees of weathering of joints lead to smaller failure loads. Once more, it is not possible to determine the weathering degree before failure. However, it was possible to show that rebound values around the anchor position correlate with the degree of weathering. A high degree of weathering leads to low rebound values and low joint weathering leads to high rebound values. Therefore, rebound values provide the possibility of making an estimation about base material quality in advance of failure. Accordingly, high rebound values indicate good base material quality. From the investigated rebound values, a threshold can be calculated based on a log-normal distribution. If rebound values around optically undisturbed anchor positions do not fall below this threshold, good base material properties can be assumed. If the criterion is met in areas of optically disturbed anchor positions, average base material quality can be assumed. This procedure should therefore always be combined with a visual assessment. Average base material can also be considered if the threshold is not met in undisturbed area. Finally, poor base material should be assumed when the criterion is not met in disturbed areas.

This technical paper was able to demonstrate the influence of joint quantity and weathering next to the anchor position on post-installed anchors in rock. Further, an approach for a preliminary assessment to classify the base material quality was proposed. An open question remains concerning the extent to which t a design concept for the base material classes can be derived from this and this will be the subject of future research. A detailed investigation of these questions helps engineers of post-installed anchors to become more familiar with the less known base material rock.

## Figures and Tables

**Table 1:** Overview of the conducted test program.

	Granulite	Dolomitic	Dolomite	Granite
		Limestone		
Location	west of Vienna, Austria	east of Vienna, Austria	southwest of Vienna, Austria	northwest of Vienna, Austria
Geology, description of base material	metamorphic	sedimentary	sedimentary	metamorphic
	narrowly fractured, fine- to medium-grained components	tectonically strongly utilized, crystallized joints filled with calcite layers	tectonically strongly utilized, narrow joint distance (centimeters to meters), joints filled with sand and clay	tectonically utilized, medium-grained metagranite
	[10]	[11,12]	[13,14]	[15,16]
Geological tests performed per location: point load test, rebound hammer, compressive strength (cylinder), Geological Strength Index (GSI), Rock Mass Rating (RMR)				
**Number of test anchors** **(h_ef_ = 70 mm, optically evaluated)**				
Disturbed area	15	15	10 ^(2)^	15 ^(2)^
Undisturbed area	15	15	- ^(1)^	15
Assessments per installation point: rebound hammer, failure load (F_u_), failure mode, joint condition (weathering), joint quantity				

^(1)^ due to rock properties not possible as no undisturbed areas have been observed; ^(2)^ two times setting failure occurred.

**Table 2:** Overview–geological assessment.

	UCS	UCS	Uniaxial	Geological	Rock Mass
Rock Type	Point Load	Schmidt	Cylindrical	Strength	Rating
	Index	Hammer	Compressive Strength	Index	
	(N/mm^2^)	(N/mm^2^)	(N/mm^2^)	in acc. to [18]	in acc. to [19]
Granulite	93.5	59.0	120.1 (x̅, *n* = 3)	52–58	76.8
Dolomitic limestone	82.8	61.0	57.5 (x̅, *n* = 2)	42–47	82.6
Dolomite	38.4	13.0	28.5 (from test report)	25–29	54.0
Granite	103.7	58.7	78.7 (x̅, *n* = 3)	54–59	86.2

**Table 3:** Comparison of failure loads for the different rock types for disturbed and undisturbed areas.

Parameter	Unit	Granulite	Limestone	Dolomite	Granite
F_u,m_	kN	32.2	38.1	10.5	45.4
coefficient of variation	%	58%	51%	81%	63%
F_u,m1_ disturbed fastening area	kN	20.1	28.3	10.5	25.3
coefficient of variation	%	49%	58%	81%	68%
F_u,m2_ undisturbed fastening area	kN	50.3	52.8	n.a.	65.5
coefficient of variation	%	26%	26%	n.a.	35%
Deviation undisturbed/disturbed	%	−60%	−48%	n.a.	−61%

**Table 4:** Standard deviations for Figure 4 and Figure 6.

	Granulite	Limestone	Dolomite	Granite
standard deviations Figure 4				
no weathering	n.a.	0.0	n.a.	15.0
low weathering	10.2	7.2	n.a.	2.5
moderate weathering	9.4	16.0	0.0	8.4
high weathering	1.1	0.0	3.1	0.0
standard deviations Figure 6				
no weathering	n.a.	0.0	n.a.	2.9
low weathering	4.2	3.3	n.a.	3.6
moderate weathering	14.9	2.3	0.0	4.9
high weathering	13.8	0.0	9.0	0.0

**Table 5:** Base material classification.

Base Material Quality		Poor	Average (1)	Average (2)	Good
rebound values per anchor >36		no	yes	no	yes
disturbed/undisturbed	disturbed		disturbed	undisturbed	undisturbed
mean Fu	/in kN	21.20	34.96	41.37	58.12
CV		69%	42%	60%	34%
minimal Fu	/in kN	3.90	18.60	7.00	25.60
